# Tai chi training reduces self-report of inattention in healthy young adults

**DOI:** 10.3389/fnhum.2014.00013

**Published:** 2014-01-27

**Authors:** Alexander K. Converse, Elizabeth O. Ahlers, Brittany G. Travers, Richard J. Davidson

**Affiliations:** ^1^Waisman Center, University of Wisconsin-MadisonMadison, WI, USA; ^2^Department of Psychology, University of Wisconsin-MadisonMadison, WI, USA

**Keywords:** Tai chi, attention deficit disorder with hyperactivity, meditation, mindfulness, non-pharmacological intervention, college students, young adults

## Abstract

It is important to identify effective non-pharmacological alternatives to stimulant medications that reduce symptoms of attention deficit hyperactivity disorder (ADHD). In this study of healthy young adults, we measured the effects of training in tai chi, which involves mindful attention to the body during movement. Using a non-randomized, controlled, parallel design, students in a 15-week introductory tai chi course (*n* = 28) and control participants (*n* = 44) were tested for ADHD indicators and cognitive function at three points over the course of the 15-weeks. The tai chi students’ self-report of attention, but not hyperactivity–impulsivity, improved compared to controls. At baseline, inattention correlated positively with reaction time variability in an affective go/no-go task across all participants, and improvements in attention correlated with reductions in reaction time variability across the tai chi students. Affective bias changed in the tai chi students, as reaction times to positive- and negative-valenced words equalized over time. These results converge to suggest that tai chi training may help improve attention in healthy young adults. Further studies are needed to confirm these results and to evaluate tai chi as therapy for individuals with ADHD.

## INTRODUCTION

The use of mind-body techniques to enhance cognitive function in young adults and adolescents would provide an attractive alternative to pharmacological treatment of conditions such as attention deficit hyperactivity disorder (ADHD), as well as non-medical use of stimulants for performance enhancement (Sussman et al., 2006; Krisanaprakornkit et al., 2010). Tai chi training may provide cognitive benefits to younger individuals as it has been shown to improve cognitive function in the elderly (Matthews and Williams, 2008; Man et al., 2010; Taylor-Piliae et al., 2010; Lam et al., 2012; Mortimer et al., 2012; Nguyen and Kruse, 2012; Lu et al., 2013). In this study we examine the effects of tai chi training on cognitive function in young adults.

Tai chi involves mindful attention to the body during a well-defined series of slow-flowing movements (Kauz, 1974; Jou, 1980). It is generally recognized as a safe and low-cost complementary therapy and is practiced by two million Americans for a variety of purposes (Barnes, 2004; Birdee et al., 2009). Recently, rigorous scientific methods have been applied to the study of the biomedical aspects of tai chi. While the purported health benefits of tai chi include psychological components, e.g., the cultivation of a state of relaxed attention, the majority of scientific studies focus on physical outcomes such as gait, posture, and cardiovascular health. These have largely examined middle-aged and elderly subjects (Wang et al., 2004a).

If tai chi were shown to improve attention in healthy subjects, it would provide support for future work to assess tai chi’s efficacy as therapy for individuals with ADHD. ADHD affects 3–7% of children in the U.S. and persists into adulthood in 30–70% of cases (American Psychiatric Association, 2000; Lara et al., 2009). More than three million American patients with ADHD are treated with the stimulants amphetamine and methylphenidate, both of which target the dopamine transporter (Levy, 1991; Swanson et al., 2007). Brain imaging studies also suggest the dopamine system plays a role in ADHD (Durston, 2010). Medications are in some cases ineffective, poorly tolerated, or not desired (Kooij et al., 2010; Castells et al., 2011; Green and Rabiner, 2012). Therefore, non-pharmacological therapies are the subject of research (Toplak et al., 2008; Zylowska et al., 2008; Knouse and Safren, 2010; Krisanaprakornkit et al., 2010; Young and Amarasinghe, 2010). In a non-controlled study of 13 adolescent individuals with ADHD, teacher ratings of symptoms improved following a 5-week tai chi course (Hernandez-Reif et al., 2001). Moreover, teenagers who struggle with ADHD-like symptoms but do not meet full criteria for ADHD have been shown to be at increased risk for several psychiatric disorders (Malmberg et al., 2011), suggesting that non-pharmacological interventions for inattention and hyperactivity in healthy individuals may also be warranted.

Here we report an observational study comparing healthy young adults undergoing 15 weeks of tai chi training to a passive control group. The outcome measures were cognitive function, physical balance, and ADHD indicators, all measured at the beginning, middle, and end of the semester. Because spatial working memory (SWM) and response inhibition are associated with ADHD and respond to stimulant therapy, our primary *a priori* hypothesis was that, compared to controls, subjects in the tai chi course would show improvements in specific measures within these neurocognitive domains (Chamberlain et al., 2011). Given reports that ADHD patients exhibit greater reaction time (RT) variability (Tamm et al., 2012; Kofler et al., 2013), in *post hoc* analyses we examined correlations between RT variability and ADHD measures.

## MATERIALS AND METHODS

### SUBJECTS

Tai chi students were recruited from the University of Wisconsin-Madison course “Introduction to Martial Arts: Tai Chi” and were compensated $30. Control subjects were recruited from the UW course “Introduction to Psychology” and were compensated with course extra credit. Subjects were required to be between the age of 18 and 34 years, and there were no exclusion criteria. Recruitment and retention details are shown in **Table 1**, and participant demographics are presented in **Table 2**. The tai chi students were older than the control subjects (24.1 ± 3.5 vs. 19.4 ± 1.3 years), but otherwise there were no significant differences. All procedures were approved by the UW Social and Behavioral Sciences Institutional Review Board (SE-2012-0539), and the study was registered with ClinicalTrials.gov as a non-randomized trial (NCT01681082).

**Table 1:** Recruitment and retention.

Number participating	Control subjects	Tai Chi students
Session 1	57	34
1 and 2 and 3	40	26
1 and 2 (not 3)	4	1
1 and 3 (not 2)	0	2
Included in analysis^a^	44 (77%)	28 (82%)

a Participants were included in the analysis if they participated in test session 1 and at least one additional test session (2 or 3). One of the two tai chi students who participated at session 3 but not at session 2 provided incomplete ASRS data at session 3 and was therefore excluded from the analysis. Percentage indicates retention, i.e., number included in analysis/number participating in session 1.

**Table 2:** Participant demographics.

	Control subjects	Tai Chi students	*p*^e^
*n*	44	28	
Sex (Female)	31 (70%)	16 (57%)	0.367
Age (mean ± SD)^a^	19.36 ± 1.27	24.14 ± 3.46	<0.001
ESL^b^	14 (32%)	6 (21%)	0.490
Mind-body^c^	17 (39%)	13 (46%)	0.683
Exercise (mean ± SD)^d^	51.2 ± 26.1	50.8 ± 35.6	0.952

*^a^At start of semester, ^b^number of subjects reporting English as second language, ^c^number of subjects reporting previous mind-body experience (mindfulness, meditation, yoga, etc.), ^d^Godin leisure time questionnaire Weekly Leisure Activity Score. ^e^*p* value for group difference, two sample *t*-test for age and exercise and chi-squared test for sex, ESL, and mind-body.*

### INTERVENTION

Tai chi students attended 50 m classes twice per week for 15 weeks, with approximately 20 students in each class. The course emphasized experiential learning with three weeks of introductory sessions on gait, posture, and tai chi principles followed by instruction in the 24-form Yang style sequence (Qu, 1999). The course has been taught for over 10 years by the same instructor, who emphasizes the mindfulness aspect of tai chi. The instructor checked attendance at each class and after a fourth absence, the final grade was lowered by one-half grade for each additional absence. Control subjects were given no training or instructions.

### PROTOCOL

All subjects underwent 1-h test sessions at the beginning, middle, and end of the 15-week semester to assess balance, cognitive function, and ADHD indicators. Testing was performed on one set of tai chi students and controls during Fall 2012 and on a second set during Spring 2013.

### BALANCE MEASURE

Subjects performed the One-Legged Stance Test (OLST), in which they stood on one leg with eyes closed as long as possible for up to 60 s per trial (Briggs et al., 1989). The test was repeated alternately on both legs three times. The average of the best time on each leg was chosen *a priori* as the outcome measure.

### COGNITIVE MEASURES

Participants performed three CANTAB^®^ (Cambridge) computer button box- and touchscreen-based tests [CANTABeclipse(TM), 2012]. In the SWM test participants search for a token in a group of up to eight boxes without returning to boxes where a token had been found in a previous trial. In the Stop Signal Task (SST) subjects are instructed to rapidly press a left or right button depending on the direction of a stimulus arrow. On a subset of trials, an auditory stop signal indicates that the subject must inhibit their response. The presentation time of the stop signal is adjusted over the course of the test so the participant is able to withhold a button press in half of the stop trials. In the Affective Go/No-Go test (AGN) the participant is informed of both the target and distractor valence (positive, negative, or neutral) for a rapidly presented series of words and instructed to press a button for words of the target valence only. Outcome measures identified *a priori* were SWM “between errors” (primary outcome measure: number of times the subject revisited a box in which a token was previously found, i.e., “between” trials with the same pattern of boxes), SST stop signal RT (a response inhibition measure: mean go-trial RT minus mean stop signal presentation time, so lower values are better), and AGN correct RT (average over positive, neutral, and negative valenced words). Additional measures included affective bias (AGN correct RT, positive minus negative valenced words), AGN RT variability (mean over three valences of SD of correct response RT), and SST RT variability (SD of RT on go trials).

### SELF-REPORT

Participants responded to four questionnaires: (1) Demographics: date of birth, sex, primary language, academic year and major, ethnicity, and race (first session only). (2) Adult ADHD Self-Report Scale (ASRS): 18 DSM-IV criteria as self-report Likert scale (0–4) items (Kessler et al., 2005). Of *a priori* interest was the sum score (0–24) from the six-question ASRS short screen consisting of inattention items 4, 5, 6, and 9 and hyperactivity–impulsivity items 1 and 5. In exploratory analysis, the two sub-scores (0–36) were evaluated for all nine inattention items and all nine hyperactivity–impulsivity items. (3) Experience with Mind-Body Practices: mindfulness, meditation, yoga, etc. (4) Godin Leisure-Time Exercise Questionnaire: frequency of strenuous, moderate, and mild exercise expressed as Weekly Leisure Activty Score (Godin and Shephard, 1985). In the Fall, subjects responded to paper versions of the questionnaires, while in the Spring, they responded to computer versions (Qualtrics). Additionally, the tai chi students were given a paper practice log and asked to keep a daily record of time spent practicing tai chi including class time; the log was collected at Session 2 and Session 3.

### STATISTICAL ANALYSIS

Analyses were performed in R version 2.15.1 (R Development Core Team, 2012). Dependent variables were analyzed using a linear mixed effects model (LMER) with subject as a random effect and group and session as fixed effects. Effects of tai chi training were inferred from group × session interactions. Nuisance variables of age, sex, ESL, mind-body experience, and weekly leisure activity score were also included as fixed effects. Correlations between baseline scores and between change scores were evaluated by linear regression. Results with *p* < 0.05 were considered significant, and no corrections were applied for multiple comparisons (five a priori measures and four exploratory measures).

## RESULTS

These results are based on data from 28 tai chi students and 44 control subjects. The tai chi students reported that they trained 101 ± 24 min per week. The effects of tai chi training were examined for the five quantities identified *a priori,* which provided specific measures of working memory, response inhibition, affective processing, physical balance, and the ADHD short screen. None of these measures exhibited significantly more change in the tai chi students compared to controls (**Table 3**). Changes from Session 1 to Session 3 in the affective go/no-go correct RT and the ADHD short screen were positively correlated across the tai chi students [*r*(27) = 0.511, *p* = 0.006]. There were no other correlations among changes in these five measures (remaining *p*’s > 0.147), nor among these five measures at session 1 (all *p*’s > 0.225).

**Table 3:** Effect of tai chi training – measures specified a priori.

	Control subjects			Tai Chi students			Group × Session^f^		
Mean (SEM)	Session 1	Session 2	Session 3	Session 1	Session 2	Session 3	β	*t*	*p*
Working memory^a^	17.18 (2.86)	10.00 (1.48)	8.35 (1.29)	13.82 (2.48)	9.15 (1.82)	9.44 (1.59)	4.738	1.659	0.099
Physical balance (s)^b^	30.84 (2.95)	31.82 (2.76)	34.36 (3.06)	36.86 (3.55)	45.82 (3.17)	46.63 (3.26)	5.937	1.726	0.086
Response inhibition (ms)^c^	162.59 (6.25)	149.38 (6.15)	152.56 (6.95)	162.54 (7.92)	139.64 (6.34)	146.70 (7.05)	-3.289	-0.321	0.749
Affective processing (ms)^d^	493.88 (12.17)	510.09 (11.20)	520.30 (10.92)	523.58 (12.50)	539.41 (10.68)	538.30 (11.72)	-8.16	-0.928	0.355
ADHD short screen^e^	8.98 (0.47)	9.61 (0.55)	9.40 (0.52)	8.54 (0.53)	8.93 (0.53)	9.15 (0.43)	0.221	0.379	0.705
Day of semester,	15.6 (7.4)	52.4 (5.8)	95.9 (3.8)	9.8 (5.8)	51.2 (7.5)	96.2 (3.9)			
Mean (SD)									

*Outcome measures identified *a priori* (clinicaltrials.gov NCT01681082). Measures from test sessions conducted at (1) beginning, (2) middle, and (3) end of semester. There were no significant differences between groups at baseline. ^a^CANTAB spatial working memory task “between errors” (primary outcome measure), ^b^One legged stance test, average across both legs of best trial, ^c^CANTAB stop signal reaction time, ^d^CANTAB affective go/no-go task mean correct RT, ^e^ASRS 6 item short screening scale, ^f^linear mixed effects model group × session interaction: β = change from session 1 to session 3 in tai chi students relative to controls, *t* = *t* value, *p* = 2-tailed *p* value.*

**FIGURE 1:**
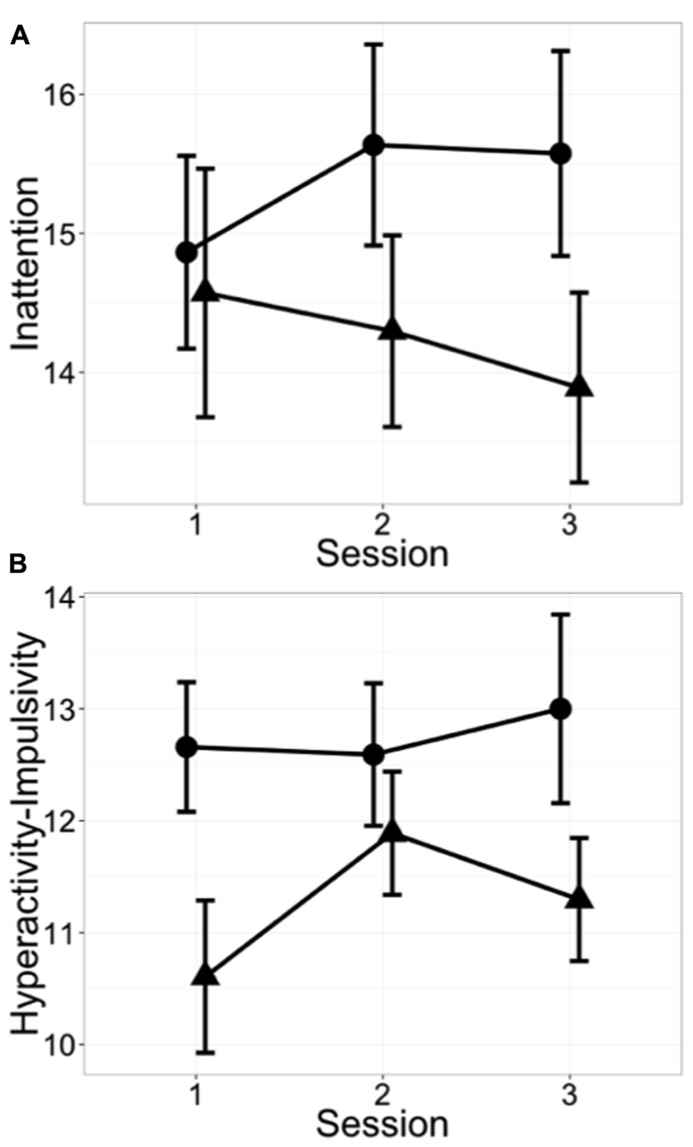
**Inattention, but not hyperactivity–impulsivity, was reduced in tai chi students compared to control subjects. (A)** ADHD indicators of inattention (ASRS inattention items 1–9) improved in tai chi students relative to controls (*p* = 0.044, linear mixed effects model group × session interaction). **(B)** No significant change was seen in hyperactivity–impulsivity (ASRS hyperactivity–impulsivity items 1–9). Test sessions conducted at (1) beginning, (2) middle, and (3) end of semester. • = control subjects; ▲ = tai chi students. Mean ± SEM.

**FIGURE 2:**
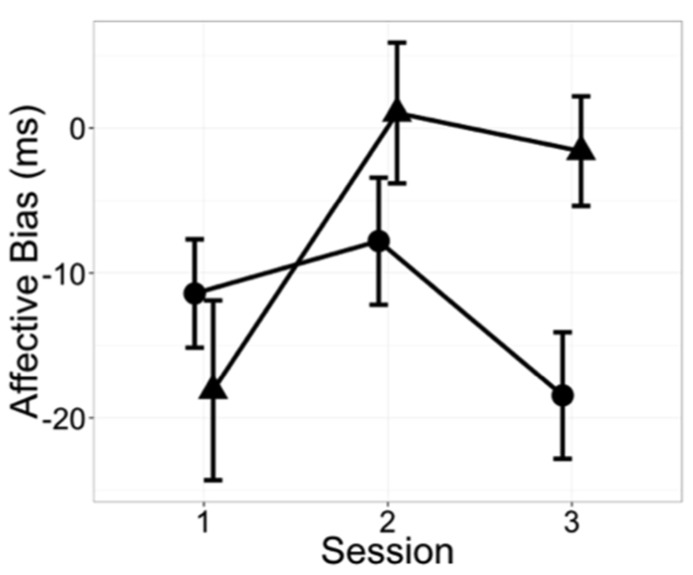
**Affective bias changed in tai chi students compared to control subjects.** Specifically, reaction times to positive and negative valenced words tended to equalize in tai chi students over time (*p* = 0.008, linear mixed effects model group x session interaction). Test sessions conducted at (1) beginning, (2) middle, and (3) end of semester. • = control subjects; ▲ = tai chi students. Mean ± SEM. Bias = positive valence reaction time–negative valence reaction time.

Given the observed correlation in changes in the affective go/no-go correct RT and the ADHD short screen in the tai chi students, additional ADHD and affective go/no-go measures were explored (**Table 4**). As shown in **Figure 1**, inattention decreased by 10% relative to controls (*p* = 0.044, group × session interaction, no group difference at baseline), but there was no significant change in hyperactivity–impulsivity (*p* = 0.748). Analysis of the less attentive subjects in each group (median split at session 1, no group difference at baseline, data not shown) yielded a 16% reduction in the tai chi students’ report of inattention compared to that seen in controls (*p* = 0.002). In the small number of participants whose baseline inattention score was above 20, the optimum threshold for concordance with clinician-rated ADHD classification (Kessler et al., 2005), tai chi students’ self-report of inattention declined by 22% compared to controls (*p* = 0.023, tai chi *n* = 3, control *n* = 4, no group difference at baseline, data not shown). Although the reduction in affective go/no-go RT variability was not significantly different between the two groups (*p* = 0.839), the numerical value of the affective go/no-go bias measure (positive–negative correct RT) increased in the tai chi students compared to controls (*p* = 0.008 **Figure 2**).

**Table 4:** Effect of tai chi training – ADHD and affective processing.

mean (SEM)	Control subjects			Tai Chi students			Group × session^f^		
	Session 1	Session 2	Session 3	Session 1	Session 2	Session 3	β	*t*	*p*
**ADHD**
Inattention^a^	14.86(0.69)	15.64(0.72)	15.57(0.74)	14.57(0.89)	14.30(0.69)	13.89(0.68)	-1.42	-2.025	0.044*
Hyperactivity–impulsivity^b^	12.66(0.58)	12.59(0.64)	13.00(0.84)	10.61(0.68)	11.89(0.55)	11.30(0.55)	0.277	0.322	0.748
**Affective processing (ms)^c^**
Bias^d^	-11.41(3.73)	-7.80(4.39)	-18.45(4.37)	-18.09(6.20)	1.03(4.85)	-1.60(3.78)	23.205	2.671	0.008*
RT variability^e^	113.59(3.80)	110.87(3.69)	111.33(3.36)	111.25(4.46)	104.99(3.68)	107.83(4.32)	-0.938	-0.203	0.839

Measures from test sessions conducted at (1) beginning, (2) middle, and (3) end of semester. There were no significant differences between groups at baseline except for hyperactivity–impulsivity. ^a^ASRS inattention items 1–9, ^b^ASRS hyperactivity–impulsivity items 1–9 (significant difference between groups at session 1, *p* = 0.027), ^c^CANTAB affective go/no-go mean correct RT, ^d^positive–negative, ^e^SD averaged over three valences, ^f^linear mixed effects model group × session interaction: β = change from session 1 to session 3 in tai chi students relative to controls, *t* = *t* value, *p* = 2-tailed *p* value.*p < 0.05.

**FIGURE 3:**
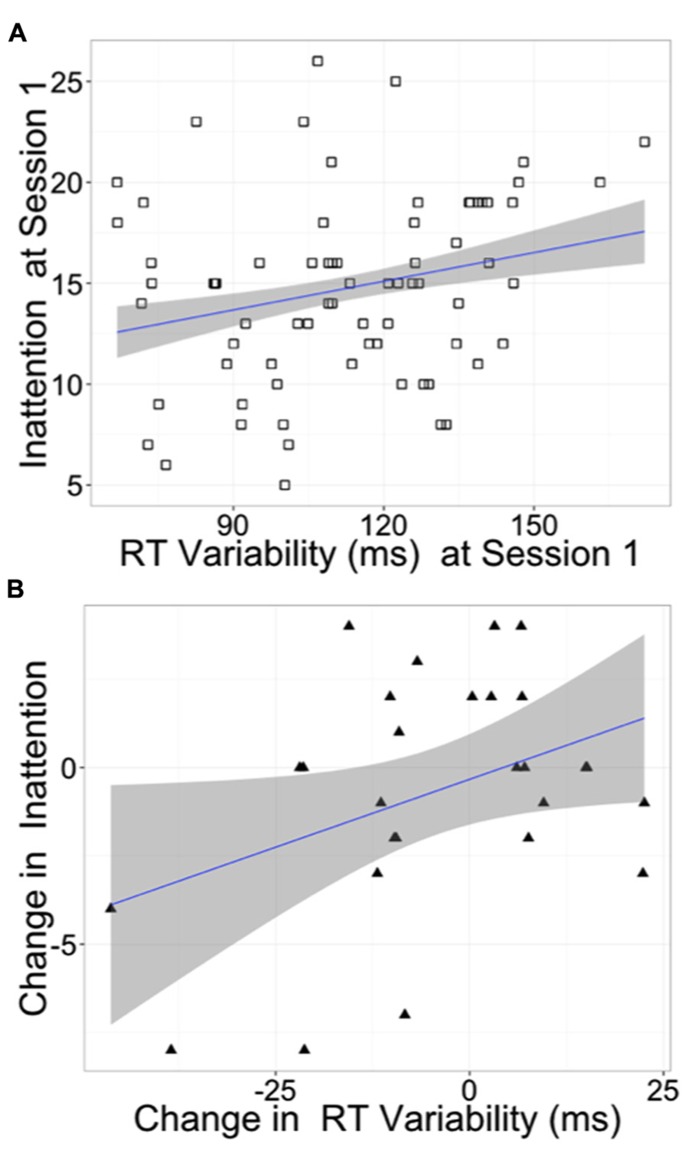
**Inattention and reaction time variability. (A)** At baseline, inattention correlated with reaction time variability in the affective go/no-go task across all subjects [*r*(72) = 0.251, *p* = 0.034], and **(B)** improvements in attention correlated with reductions in reaction time variability across the tai chi subjects [*r*(27) = 0.387, *p* = 0.046]. Change from session 1 to session 3. Inattention: ASRS inattention items 1–9. Reaction Time (RT) Variability: mean over three valences of affective go/no-go correct RT SD.

Because RT variability has been proposed as a neurocognitive marker for ADHD (Tamm et al., 2012; Kofler et al., 2013), we explored correlations between the ADHD sub-scores for inattention and for hyperactivity–impulsivity with RT variability in the SST and the affective go/no-go task (**Table 5**). As shown in **Figure 3**, at session 1, inattention correlated positively with affective go/no-go RT variability across all subjects [*r*(72) = 0.251, *p* = 0.034]. From session 1 to session 3, improvements in attention were correlated with reductions in affective go/no-go RT variability across the tai chi students [*r*(27) = 0.387, *p* = 0.046] but not across the control subjects [*r*(40) = 0.073, *p* = 0.655]. There was, however, no significant group difference between these correlations (*p* = 0.20). In addition, tai chi students who reported more practice time tended to exhibit greater reductions in affective go/no-go RT variability at a trend level [*r*(27) = -0.320, *p* = 0.104]. There were also correlations in unexpected directions of practice time with change in balance [*r*(27) = -0.372, *p* = 0.056] and change in the ADHD hyperactivity–impulsivity sub-score [*r*(27] = 0.397, *p* = 0.040].

**Table 5:** Correlations between ADHD subscores and reaction time variability: at baseline and change over time.

Pearson *r*	All subjects baseline^g^	Control Δ^h^	Tai Chi Δ^h^
**Inattention^a^ vs**
Stop signal task^c^	0.137	0.227	0.217
Affective go/no-go^d^	0.251*^e^	0.073	0.387*^f^
**Hyperactivity–impulsivity^b^ vs**
Stop signal task	0.077	-0.026	-0.175
Affective go/no-go	0.108	-0.058	-0.104
n^i^	72	40	27

^a^ASRS inattention items 1–9; ^b^ASRS hyperactivity–impulsivity items 1–9; ^c^CANTAB stop signal task, SD of reaction time over go trials; ^d^CANTAB affective go/no-go task, correct RT SD averaged over 3 valences; ^e^*p* = 0.034; ^f^*p* = 0.046; ^g^correlation between measures at session 1; ^h^correlation between changes from session 1 to session 3; ^i^session 3 data was unavailable for four control subjects and one tai chi student. *p < 0.05.

## DISCUSSION

We examined the effects of tai chi training on selected attentional and cognitive processes in healthy young adults, and found a reduction in self-reported inattention that was supported by neurocognitive measures. These results suggest tai chi training might serve as therapy for young adults and adolescents suffering from ADHD inattention symptoms. Additional results pointed to the potential sensitivity of emotional processing measures to tai chi training.

Attention deficit hyperactivity disorder inattention indicators decreased in tai chi students, and the improvements in attention correlated with reductions in RT variability in an affective go/no-go task. Reviews of the literature suggest RT variability may be a marker of ADHD (Tamm et al., 2012; Kofler et al., 2013). Indeed, in addition to the correlated changes seen in the tai chi students, affective go/no-go RT variability correlated with inattention across all subjects at baseline. These improvements in attention observed in healthy young adults lend credence to the notion that tai chi training might serve as an effective therapy for adolescents and young adults with ADHD. Encouragingly, in less attentive subsets of subjects from each group, whose attention scores were equivalent at baseline, the tai chi students exhibited greater reductions in inattention compared to controls. However, while self-report of inattention decreased significantly compared to controls, there was no significant change in self-report of hyperactivity–impulsivity. To our knowledge, the only report of tai chi as therapy for ADHD describes a single-arm study of 13 adolescent patients, in which teacher report of symptoms declined after 5 weeks of training (Hernandez-Reif et al., 2001). The present results underscore the potential of tai chi training and indicate the need for additional studies in ADHD patients.

Beyond the observed reduction in self-report of inattention and associated reductions in affective go/no-go RT variability, the present study yielded additional interesting results. Affective bias increased in the tai chi students as the rates of response to positive and negative words tended to equalize. Among the measures identified *a priori* the only significant result was the correlation in changes in the ADHD six-question screen and affective go/no-go RT. This correlation was echoed by the correlation seen between the ADHD inattention score and the affective go/no-go RT variability. Contrary to our *a priori* hypotheses, we actually observed a trend-level decline in the tai chi students’ performance of the spatial working memory task compared to controls, and no significant improvement was observed in the SST measure of response inhibition. Analyses of dose effect, i.e., correlations of change in outcome measures with practice time, present a complex picture and suggest the need for more accurate measures of time spent practicing tai chi. Physical balance improved in the tai chi subjects compared to the controls, but only at trend level. It is also noteworthy that in the stop signal task measure of response inhibition, correlations between ADHD measures and RT variability, though not significant, were in the same direction as those seen in the affective go/no-go task, i.e., reduced RT variability was associated with improved ADHD indicators. Taken together, these additional results suggest measures of affective processing may be sensitive to tai chi training.

These results contribute to a small but growing body of literature describing the effects of tai chi training in healthy young adults. This literature suggests that tai chi training may lead to improvements in self-report of physical and mental health measures (Wang et al., 2004b), decreased nightmares (Slater and Hunt, 1997), improvements in self-report of mindfulness, mood, perceived stress, and sleep quality (Caldwell et al., 2011), reductions in salivary cortisol as well as improvements in self-report of mental health measures and perceived stress (Esch et al., 2007), and improvements in measures of blood plasma immunological markers (Wang and An, 2011). Collectively, this literature suggests tai chi training in young adults may have salutary effects on mental health, perceived stress, and immune function. This literature also points to the need for more randomized controlled trials with objective measures of cognitive function.

The present study was strengthened by its interventional design, which, as opposed to a cross-sectional comparison of experienced and naive practitioners, permits inference of causality due to tai chi training. The use of a comparison group accounted for practice effects in testing. An additional strength was the use of objective neurocognitive measures. This study also had a number of limitations. The main findings resulted from exploratory analyses that do not survive multiple comparison correction, and they will require replication. It would be of particular interest to repeat the measures of inattention, RT variability, and affective bias. Although we used a control group, this was an observational study without randomization, so self-selection biases may exist. Mean age differed between the groups, although age was included as a nuisance variable in the linear mixed effects model. Because participants were aware that the purpose was to measure the psychological effects of tai chi training, demand characteristics may have influenced the results, although the reduction in self-report of ADHD indicators was supported by the objective RT variability measures. Researchers were aware of the subjects’ group status and may therefore have introduced bias in their administration of the tests. Because a single experienced teacher provided tai chi instruction, generalizability is limited. Finally, we did not assess potentially confounding medical or recreational drug use. Future studies of cognitive effects of tai chi training will ideally be randomized controlled trials with an active control intervention to balance the social and physical aspects of tai chi along with assessment of confounding variables including participant expectations.

In conclusion, the results of this study in healthy young adults suggest that tai chi training improves attention and may therefore hold potential as a non-pharmacological intervention for individuals with ADHD. Additional studies are needed to confirm these results in healthy subjects and to extend this research to ADHD patient populations.

## AUTHOR CONTRIBUTIONS

Alexander K. Converse developed the study concept. Alexander K. Converse, Elizabeth O. Ahlers, and Richard J. Davidson contributed to the study design. Testing and data collection were performed by Alexander K. Converse. Alexander K. Converse and Brittany G. Travers performed the data analysis and interpretation. Alexander K. Converse drafted the paper. Brittany G. Travers and Richard J. Davidson provided critical revisions. All authors approved the final version of the paper for submission.

## Conflict of Interest Statement

The authors declare that the research was conducted in the absence of any commercial or financial relationships that could be construed as a potential conflict of interest.
